# Breeding value prediction for production traits in layer chickens using pedigree or genomic relationships in a reduced animal model

**DOI:** 10.1186/1297-9686-43-5

**Published:** 2011-01-21

**Authors:** Anna Wolc, Chris Stricker, Jesus Arango, Petek Settar, Janet E Fulton, Neil P O'Sullivan, Rudolf Preisinger, David Habier, Rohan Fernando, Dorian J Garrick, Susan J Lamont, Jack CM Dekkers

**Affiliations:** 1 Department of Genetics and Animal Breeding, University of Life Sciences in Poznan, Wołyńska st. 33, 60-637 Poznan, Poland; 2 Department of Animal Science, Iowa State University, Ames, IA 50011-3150, USA; 3 Applied Genetics Network, Börtjstrasse 8b, 7260 Davos, Switzerland; 4 Hy-Line International, Dallas Center, IA 50063, USA; 5 Lohmann Tierzucht GmbH, 27472 Cuxhaven, Germany

## Abstract

**Background:**

Genomic selection involves breeding value estimation of selection candidates based on high-density SNP genotypes. To quantify the potential benefit of genomic selection, accuracies of estimated breeding values (EBV) obtained with different methods using pedigree or high-density SNP genotypes were evaluated and compared in a commercial layer chicken breeding line.

**Methods:**

The following traits were analyzed: egg production, egg weight, egg color, shell strength, age at sexual maturity, body weight, albumen height, and yolk weight. Predictions appropriate for early or late selection were compared. A total of 2,708 birds were genotyped for 23,356 segregating SNP, including 1,563 females with records. Phenotypes on relatives without genotypes were incorporated in the analysis (in total 13,049 production records).

The data were analyzed with a Reduced Animal Model using a relationship matrix based on pedigree data or on marker genotypes and with a Bayesian method using model averaging. Using a validation set that consisted of individuals from the generation following training, these methods were compared by correlating EBV with phenotypes corrected for fixed effects, selecting the top 30 individuals based on EBV and evaluating their mean phenotype, and by regressing phenotypes on EBV.

**Results:**

Using high-density SNP genotypes increased accuracies of EBV up to two-fold for selection at an early age and by up to 88% for selection at a later age. Accuracy increases at an early age can be mostly attributed to improved estimates of parental EBV for shell quality and egg production, while for other egg quality traits it is mostly due to improved estimates of Mendelian sampling effects. A relatively small number of markers was sufficient to explain most of the genetic variation for egg weight and body weight.

## Background

During the first decade of the 21st century, there has been a rapid development of genomic selection tools. Through the application of genomic selection [1], marker information from high-density SNP genotyping can increase prediction accuracies at a young age, shorten generation intervals and improve control of inbreeding [2], which should lead to higher genetic gain per year. Many simulation studies have shown the benefits of this technology, depending on heritability, number and distribution of effects of QTL, population structure, size of training data set used to estimate SNP effects, and other factors [3]. However, studies on real data are still scarce. If practical application of genomic selection is to be implemented in chicken breeding, as already done for dairy cattle [4], it must prove its advantage over traditional methods and be used in a way that maximizes the use of available information. The accuracy of EBV derived from large numbers of markers for within-breed selection is difficult to evaluate analytically and must be validated by correlating predictions to phenotype in the target population (usually the generation following training).

One of the challenges in genomic prediction of breeding values is that not all phenotyped individuals are genotyped. One approach to exploit all available information is to first estimate breeding values of genotyped individuals by pedigree-based methods using all data, including phenotypes on non-genotyped relatives, and then use deregressed estimates of those EBV for marker-based analyses [5,6]. This two-step approach may, however, result in suboptimal use of information. Another recently developed method uses a combined pedigree and genomic covariance matrix, which can incorporate both genotyped and non-genotyped animals [7,8]. However, these methods are computationally demanding and require careful scaling of the genomic relationship matrix to be consistent with the pedigree-based relationship matrix.

The reduced animal model was proposed by Quaas and Pollak [9] to make breeding value prediction under the animal model less computationally demanding. It fits the full relationship matrix for parents and absorbs the equations for non-parents. Nowadays, the development of powerful computers makes the reduction of computing cost less relevant for pedigree-based analyses but the reduced model can also be used to exploit marker-based relationships. In breeding programs using marker information, individuals that have been used for breeding (i.e. parents) are more likely to be genotyped than unselected non-parents. Estimating breeding values for genotyped animals and absorbing non-genotyped progeny into their equations can make full use of all available data. With this approach, there is no need to construct the inverse of the combined pedigree and genomic covariance matrix of Legarra et al. [7].

The objectives of this study were to implement a reduced animal model to estimate breeding values using high-density SNP genotypes, to evaluate the accuracy of breeding values estimated using high-density SNP genotypes in the generation following training in a layer breeding line, and to compare the accuracy of alternative methods of breeding value estimation.

## Methods

### Data

Data on nine traits collected during the first 22 weeks of production were recorded on 13,049 birds from five consecutive generations in a single brown-egg layer line: egg production (ePD, percent hen average); age at sexual maturity (eSM, d); weight of the first three eggs laid by the hen (eE3, g) and shell color (eC3) collected from same eggs by Chroma Meter that measures lightness (L) and hue (as a function of a red-green (a) and a yellow-blue (b) scale). A second set of egg quality traits collected at 26-28 weeks (early, e) included average weight of eggs (eEW,g); egg color (eCO) eggs; shell quality measured as puncture score - a non-invasive deformation test averaged over points of the shell (ePS, Newton); albumen height (eAH, mm); and yolk weight (eYW, g). For birds selected on the basis of early (e) trait data, also late (l) production (42-46 weeks of age) traits were recorded: body weight (lBW, g); egg production (lPD, percent hen average); puncture score (lPS, Newton); egg weight (lEW, g); albumen height (lAH, mm); egg color (lCO, Lab); and yolk weight (lYW, g). Early and late egg quality measurements were averages of records on three to five eggs. In total 2,708 animals were genotyped for 23,356 segregating SNP (minor allele frequency >0.025; maximum proportion of missing genotypes <0.05; maximum mismatch rate between parent-offspring pairs <0.05; parentage probability >0.95), using a custom high-density Illumina SNP panel. Of the genotyped animals, 1,563 were females with individual phenotypes and 1,145 were males without phenotypes. The genotyped set included sires and dams used for breeding in generations 1 to 5 and some progeny from generation 5. Breeding values were estimated for two stages of selection. To represent selection at a very young age, when own performances and phenotypes on female sibs were not yet available, training used all phenotypic data excluding generation 5, and validation was performed on 290 genotyped female individuals from generation 5. To represent selection of males at a later age, when phenotypes on female sibs are available, phenotypes of 2,167 non-genotyped hens from generation 5 were added to the training data but validation individuals were unchanged. A basic description of these data is given in Table 1.

**Table 1:** Description of the population in terms of the number, mean and standard deviation of phenotypes by trait and generation

Generation		ePD	eEW	ePS	eAH	eCO	eE3	eC3	eYW	eSM	lBW	lPD	lEW	lPS	lAH	lCO	lYW
	N	2,738	2,737	2,738	2,737	2,738	2,729	2,729	2,728	2,738	647	635	649	649	649	649	646
G1Training	Mean	80.93	56.81	1425	7.06	73.33	43.64	74.56	15.19	149.30	1.96	77.25	61.46	1,435	6.56	72.38	17.80
	Std	11.28	4.60	38.38	0.95	7.74	4.54	7.92	1.12	7.42	0.25	12.07	4.60	24.96	0.87	7.64	1.21
	N	2,772	2,772	2,770	2,771	2,771	2,752	2,753	2,736	2,772	793	784	794	794	794	794	793
G2Training	Mean	82.39	57.48	1388	7.50	71.37	46.72	74.41	15.12	156.34	1.97	80.55	62.22	1,400	7.21	66.87	17.78
	Std	11.30	4.76	39.88	1.02	8.19	5.13	7.68	1.13	9.89	0.23	12.11	4.50	40.60	0.91	9.28	1.31
	N	2,965	2,964	2,964	2,963	2,964	2,951	2,952	2,958	2,964	781	778	782	782	782	782	781
G3Training	Mean	84.85	57.92	1495	7.41	76.11	47.33	75.43	15.31	159.81	1.95	82.36	63.52	1,509	7.19	72.89	18.14
	Std	9.77	4.85	42.52	1.03	7.52	4.64	7.85	1.15	6.21	0.25	11.00	4.66	36.38	0.90	7.90	1.35
	N	2,117	2,117	2,115	2,116	2,117	2,103	2,103	2,115	2,117	759	755	768	769	769	769	768
G4Training	Mean	83.32	57.20	1460	7.37	77.15	45.22	78.10	15.10	147.57	1.77	80.02	62.65	1,496	6.87	70.93	18.09
	Std	10.28	4.92	42.79	0.98	7.72	4.74	7.86	1.23	7.82	0.27	11.02	4.77	36.61	0.94	8.59	1.38
	N	2,167	2,167	2,164	2,167	2,167	2,157	2,158	2,164	2,167	768	769	772	772	771	772	769
G5Training	Mean	85.99	58.59	1486	8.06	78.70	47.38	79.38	15.20	155.33	1.81	82.90	62.66	1,477	7.65	72.71	17.88
	Std	9.55	4.93	46.84	1.01	8.16	4.96	7.59	1.20	8.80	0.25	10.01	4.67	36.53	0.89	9.08	1.41

	N	290	290	289	290	290	278	278	290	290	277	274	280	280	280	280	275
G5Validation	Mean	83.09	59.17	1,493	7.70	78.06	45.02	80.19	15.38	148.89	1.80	77.38	63.31	1,488	7.47	71.55	17.92
	Std	9.20	4.78	41.74	1.09	7.29	4.53	7.56	1.10	7.84	0.27	11.70	4.93	35.01	0.93	8.58	1.38

Early (e) traits recorded at 26-28 weeks of life: egg production (ePD); age at sexual maturity (eSM); shell quality (ePS); weight of first 3 eggs (eE3); color of first 3 eggs (eC3); egg weight (eEW); albumen height (eAH); egg color (eCO); and yolk weight (eYW); late (l) traits recorded at 42-46 weeks: body weight (lBW); egg production (lPD); egg weight (lEW); albumen height (lAH); egg color (lCO); and yolk weight (lYW)

### Statistical analysis

Because of the data structure, a reduced animal model was applied with all parents genotyped and many non-genotyped non-parent progeny with phenotypes. In this approach, a distinction is made between genotyped individuals, including all parents, for which the full relationship matrix is fitted, and non-genotyped non-parent individuals. The following model was applied, following White et al. [10]:

$$y=Xb+(P+\frac{1}{2}QS+\frac{1}{2}QD)a+e$$

where

**y **is the (*N*x1) vector of observations,

**b **is the (25 × 1) vector of generation-hatch-line fixed effects,

**X **is the (*N*x25) incidence matrix for fixed effects,

**a **is the (*p*x1) vector of breeding values of genotyped individuals, with variance-covariance matrix **G **$\sigma_{a}^{2}$,

**P **is the (*N *× *p*) matrix with element *ij *= 1 if the *i*th observation is on genotyped individual *j*, zero otherwise,

**Q **is an (*N *× *N*) diagonal matrix with element *ii *= 1 if observation *i *is on a non-genotyped individual, zero otherwise,

**S **and **D **are (*N *× *p*) incidence matrices with elements in rows for non-genotyped individuals that correspond to the columns identifying sires and dams set to 1, and zero's elsewhere.

**e **is the (Nx1) vector of random errors which has variance $\sigma_{e}^{2}$ for observations on genotyped individuals and $\sigma_{e}^{2}+\frac{1}{2}\sigma_{a}^{2}$ for observations on non-genotyped individuals, ignoring the effect of parental inbreeding on Mendelian sampling variance in progeny.

Population size and avoiding the mating of close relatives insured low inbreeding in this population. Furthermore, variance component estimates from a full animal model and the reduced animal model described above, using pedigree relationships, were very close. Thus, ignoring the effect of parental inbreeding on Mendelian sampling variance in progeny is expected to have a negligible impact on results.

Three models were used to predict breeding values of individuals in generation 5:

1) PBLUP - Reduced animal model using pedigree relationships.

2) GBLUP - Reduced animal model using marker-based relationships for genotyped birds, with covariance matrix derived by the method of VanRaden [11], using allele frequencies based on all genotyped animals.

3) Bayes-C-π - A genomic prediction method similar to Bayes-B of Meuwissen et al. [1], except for the estimation of the proportion of SNP with zero effects (π) and assuming a common variance for all fitted SNP, with a scaled inverse chi-square prior with *ν*_*a *_degrees of freedom and scale parameter $S_{a}^{2}$, as described by Habier et al. [12]. The prior for π was uniform (0,1). The chain length was 160,000 iterations, with the first 50,000 excluded as the burn in period. In this analysis, the average genotype (number of 'B' vs. 'A' alleles) of the genotyped parents was used to fit SNP genotype effects to the pre-adjusted mean performance of their non-genotyped progeny. To account for different residual variances for progeny means, residual variances were scaled using weights derived from $w_{p}=\frac{1-h^{2}}{(1-0.5h^{2})/p}$, where *p *is the number of phenotypes included in the mean [5].

All models included the fixed effect of hatch within generation, either fitting it in the model (for PBLUP and GBLUP) or pre-adjusting the data by subtracting solutions from a single trait animal model that included all observations and pedigree relationships (for Bayes-C-π). The PBLUP and GBLUP analyses were performed using ASREML [13] and Bayes-C-π using GenSel [12]. The correlation between EBV with hatch-corrected phenotype (as described above) in the validation data sets divided by square root of heritability and regression of hatch-corrected phenotype on EBV were used as measures of accuracy and bias of EBV, respectively. Another comparison of methods was based on selecting the top 30 individuals from the 290 available for validation based on EBV for each trait and comparing the average hatch-corrected phenotype of the selected individuals. Marker based parental average (PA) EBV were also calculated for animals in the validation sets to evaluate the extent to which improvements in accuracy with use of markers resulted from more accurate estimates of Mendelian sampling terms versus more accurate EBV of the parents. This was possible in this population because parents of both sexes were genotyped. To check if combining marker-based estimates with PA increases accuracies of estimates, as suggested by VanRaden et al. [6] for dairy cattle, linear regression of pre-adjusted phenotypes on PA and genomic EBV was performed; if GEBV capture all pedigree information, then adding PA to the regression model is not expected to increase the ability to predict phenotype in validation animals.

## Results and discussion

Estimates of heritability from single-trait pedigree-based animal models fitted to the whole data set are shown in Table 2. Estimates were low to moderate for production and shell quality and moderate to high for all other egg quality traits, as expected. Estimates of heritability for early traits were higher than for the corresponding late traits. Variance components for the late traits may be biased because only selected birds had the opportunity to obtain phenotypes for these traits.

**Table 2:** Estimates of heritability from single-trait pedigree-based animal model analyses for early (e) traits recorded at 26-28 weeks of life and for late (l) traits recorded at 42-46 weeks

	Early traits							
Trait	ePD	eEW	ePS	eAH	eCO	eYW	eE3	eC3
h^2^	0.39	0.74	0.29	0.55	0.72	0.47	0.64	0.66

	**Late traits**							

Trait	lPD	lEW	lPS	lAH	lCO	lYW	lBW	eSM
h^2^	0.26	0.67	0.25	0.52	0.67	0.50	0.48	0.55

^1 ^Standard errors of heritability were between 0.02 and 0.03; early (e) traits recorded at 26-28 weeks of life: egg production (ePD); age at sexual maturity (eSM); shell quality (ePS); weight of first 3 eggs (eE3); color of first 3 eggs (eC3); egg weight (eEW); albumen height (eAH); egg color (eCO); and yolk weight (eYW); late (l) traits recorded at 42-46 weeks: body weight (lBW); egg production (lPD); egg weight (lEW); albumen height (lAH); egg color (lCO); and yolk weight (lYW)

### Accuracy of marker-based EBV

**Figure 1:**
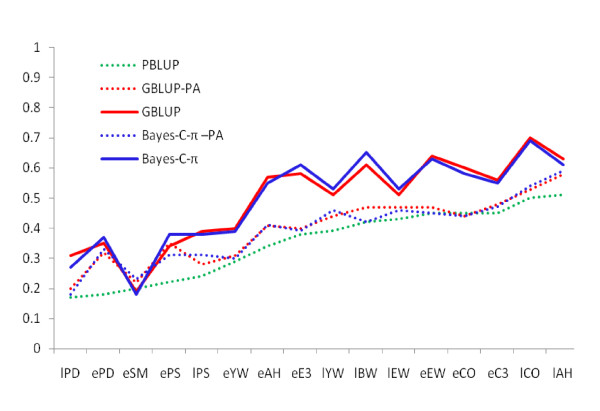
**Accuracy of predicted breeding values and parental average (PA) breeding values from three methods: pedigree-based BLUP (PBLUP), marker-based BLUP (GBLUP), and Bayesian variable selection prediction (Bayes-C-π) in the early selection scenario**. Accuracy is the correlation between predicted breeding values and hatch-corrected phenotype in the validation set divided by square root of heritability from Table 2.

**Figure 2:**
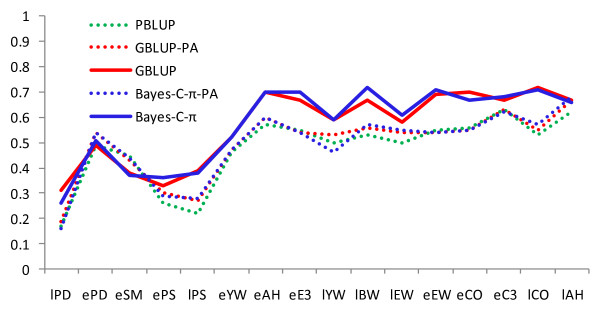
**Accuracy of predicted breeding values and parental average (PA) breeding values from three methods: pedigree-based BLUP (PBLUP), marker-based BLUP (GBLUP), and Bayesian variable selection prediction (Bayes-C-π) in the late selection scenario**. Accuracy is the correlation between predicted breeding values and hatch-corrected phenotype in the validation set divided by square root of heritability from Table 2.

**Figure 3:**
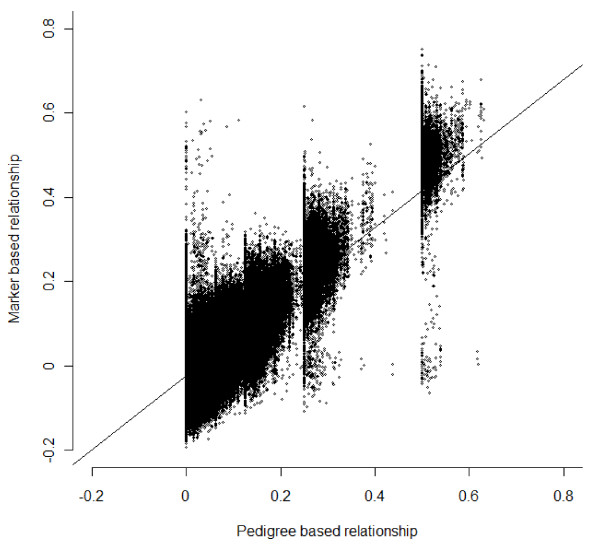
**Pedigree and marker based relationships in the studied population**.

Marker-based EBV had, in general, a higher predictive ability than estimates using pedigree relationships (Figures 1 and 2) for all traits and for early and late selection scenarios. The advantage of GBLUP over PBLUP is due to the fact that realized marker-based genetic similarity between animals deviate from pedigree-based relationship coefficients. In addition, marker-based EBV are not affected by pedigree errors, although they are affected by genotyping errors and errors in DNA sample identification. As shown in Figure 3, marker-based relationships varied substantially around pedigree relationships. The regression of marker-based on pedigree-based relationships was 0.88 for all individuals and 0.97 for validation individuals, demonstrating on average good agreement between both types of relationships. The correlation between the two relationship measures was 0.68 and 0.72 for all and validation individuals, respectively.

The difference in accuracy between GBLUP and PBLUP was smaller for selection at a later age than at an early age, when data on sibs of selection candidates were available (Figures 1 and 2). This extra information increased the accuracy of all methods and particularly of PBLUP. Using marker-based relationships increased accuracies up to over two-fold for early selection and by up to 88% for late selection. Proportionally, the highest gain in accuracy was achieved for traits with the lowest heritability. Accuracies obtained with GBLUP were on average slightly larger than those with Bayes-C-π. Several simulation studies have shown that the accuracy of Bayesian methods is higher than that of GBLUP [1,14,15] but a simulation study reported by Daetwyler et al. [16] has shown that the relative performance of GBLUP depends to a large extent on the genetic architecture of the trait. Also, studies on real data in dairy cattle have shown that GBLUP can be equally accurate or even superior in prediction for traits for which no individual QTL explains a large proportion of the variation [17,18].

Correlations for selection at an early age between EBV obtained by PBLUP and GBLUP ranged from 0.48 to 0.70 across the traits; from 0.46 to 0.71 between EBV from PBLUP and Bayes-C-π; and from 0.79 to 0.97 between EBV from GBLUP and Bayes-C-π. This indicates that reranking of top individuals is very likely between pedigree- and marker-based methods but limited between GBLUP and Bayes-C-π. This was confirmed by the average performance of the top 30 individuals selected with different methods (Table 3), which was similar for marker-based methods but somewhat different for the group selected based on pedigree EBV. A similar tendency was observed for ranking at late selection but correlations between EBV from different methods were higher for this scenario.

**Table 3:** Validation of predicted breeding values and parental average (PA) breeding values from three methods: pedigree-based BLUP (PBLUP), marker-based BLUP (GBLUP), and Bayesian variable selection prediction (Bayes-C-π), for early and late selection

Method	ePD	eEW	ePS	eAH	eCO	eE3	eC3	eYW	**eSM**^**1**^	**lBW**^**1**^	lPD	lEW	lPS	lAH	lCO	lYW
EARLY SELECTION																

Slope from regression of phenotype on EBV																

PBLUP	0.63	1.12	0.71	0.87	0.93	0.88	0.85	0.70	0.56	1.06	0.52	1.05	0.56	1.16	1.03	0.88
GBLUP	0.53	0.87	0.58	0.93	0.70	0.81	0.67	0.58	0.34	1.07	0.54	0.73	0.61	1.05	0.93	0.82
Bayes-C-π	0.65	0.93	0.68	0.94	0.69	0.86	0.73	0.59	0.34	1.01	0.56	0.91	0.72	1.13	0.98	0.91

Average performance of top 30 individuals																

PBLUP	89.9	61.0	1459.3	7.78	84.2	46.0	82.2	15.4	148.0	1.78	80.8	65.1	1453.3	7.29	76.5	18.1

GBLUP	90.3	63.5	1452.4	8.38	86.8	47.4	83.3	15.5	147.3	1.73	79.9	65.1	1440.9	7.22	80.0	18.3

Bayes-C-π	91.2	62.0	1453.7	8.41	85.9	48.3	83.4	15.4	147.2	1.70	78.1	64.7	1449.9	7.12	80.3	18.3

LATE SELECTION																

Slope from regression of phenotype on EBV																

PBLUP	1.08	0.90	0.51	1.13	0.93	0.85	1.07	0.80	0.90	1.12	0.47	1.04	0.46	1.29	0.95	0.97
GBLUP	0.72	0.85	0.50	1.06	0.82	0.83	0.81	0.72	0.60	1.10	0.54	0.83	0.60	1.06	0.91	0.89
Bayes-C-π	0.81	0.93	0.57	1.10	0.80	0.89	0.86	0.75	0.65	1.06	0.51	1.01	0.69	1.13	0.97	0.97

^1^low values are desired

The presence of bias in EBV was evaluated by regressing phenotypes of validation individuals on their EBV. On average, these regression coefficients tended to be lower than the expected value of 1: 0.9 for PBLUP, 0.8 for GBLUP and 0.86 for Bayes-C-π, which suggests that EBV overestimated differences in phenotypes of progeny. This bias may be due to selection not being properly accounted for by the single-trait analyses or due to the assumption of normality for genotypic values not being valid. However, the mean squared deviation of the regression coefficients from 1 was lowest for Bayes-C-π, 0.05, compared to 0.06 for PBLUP and 0.07 for GBLUP, suggesting that estimates from Bayes-C- π were least biased. Sib information tended to improve the performance of all methods in this regard for most traits.

### Estimation of π, the proportion of markers with zero effects

The proportion of markers with zero effect (π) is estimated from the data in the Bayes-C-π method. Habier et al. [12] have shown that, if there is enough information in the data, (1- $\hat{\pi }$)k is a good estimate of the number of QTL affecting the trait when k unlinked SNP with normally distributed effects were simulated and genotypes used for training included genotypes at the QTL. In the case of more realistic simulations, where QTL genotypes were not included as markers but the effects were estimated based on k linked markers, the number of markers fitted was higher than the number of true QTL, but the tendency for lower estimates of π for scenarios with more QTL did hold [12].

The posterior means of π (Table 4) suggest that a high proportion of markers should be included in the model to explain a substantial part of the genetic variation for the majority of traits in our data; estimates of π ranged from 0.19 to 0.99, which suggests that between 111 and 19,541 markers explained variation for the analyzed traits (Table 4). The large number of associated markers with relatively small effects explains the good performance of GBLUP, which assumes a polygenic determination of traits. However, GBLUP also performed well for egg weight and body weight, which had very high estimates of π. The results suggest that a limited number of markers explain most of the genetic variation for body size in chickens. This can be due to these markers being linked to or in linkage disequilibrium with QTL and/or due to markers capturing pedigree relationships [19].

**Table 4:** Estimates of the proportion of markers with zero effects (x100 ± SD) from the Bayesian variable selection model with starting values of 0.1 (π = 0.1) or 0.99 (π = 0.99)

	Early selection							
Trait	ePD	eEW	ePS	eAH	eCO	eYW	eE3	eC3
π = 0.1 ± SD	34 ± 19	98 ± 0	33 ± 20	42 ± 32	90 ± 5	60 ± 29	98 ± 0	48 ± 29
π = 0.99 ± SD	21 ± 21	98 ± 0	58 ± 27	71 ± 20	91 ± 3	45 ± 25	98 ± 1	60 ± 26

Trait	lPD	lEW	lPS	lAH	lCO	lYW	lBW	eSM
π = 0.1 ± SD	19 ± 17	99 ± 0	42 ± 27	37 ± 31	38 ± 23	36 ± 16	99 ± 3	33 ± 24
π = 0.99 ± SD	34 ± 25	99 ± 0	49 ± 30	30 ± 21	56 ± 30	90 ± 9	99 ± 3	58 ± 27

	**Late selection**							

Trait	ePD	eEW	ePS	eAH	eCO	eYW	eE3	eC3
π = 0.1 ± SD	38 ± 28	98 ± 0	40 ± 22	82 ± 9	92 ± 4	64 ± 24	97 ± 1	69 ± 16
π = 0.99 ± SD	36 ± 21	98 ± 1	35 ± 22	51 ± 29	92 ± 3	39 ± 20	97 ± 1	52 ± 33

Trait	lPD	lEW	lPS	lAH	lCO	lYW	lBW	eSM
π = 0.1 ± SD	36 ± 17	98 ± 0	41 ± 24	59 ± 26	40 ± 22	43 ± 32	99 ± 2	48 ± 23
π = 0.99 ± SD	49 ± 31	98 ± 1	24 ± 21	41 ± 27	45 ± 20	57 ± 28	99 ± 2	32 ± 19

The accuracy of estimates of π depends on the information content of the data and on mixing in the Monte Carlo Markov Chain, which can be poor for Bayes-C- π. Two independent chains with a high (0.99) or a low (0.1) starting value for π were used to verify convergence of π. For some traits (eE3, eEW, lEW, eCO, lBW), both chains converged to the same value with a clearly peaked posterior distribution but for other traits 160,000 iterations were not sufficient for the two chains to reach the same posterior means, as the posterior distribution of π was relatively flat. This difference may reflect differences in genetic architecture of the traits. For traits with a high estimate of π (i.e. with few markers associated), convergence was obtained quickly and the standard deviation of the posterior distribution of π was small but for traits for which many markers were fitted in the model, the standard deviation of π was high, which suggests that models with different numbers of markers had similar likelihoods. Nevertheless, lack of convergence in π, i.e. different estimates depending on starting value, had almost no impact on the accuracy of EBV. There was also no substantial difference between early and late selection scenarios with regard to convergence of estimates of π. Only for ePD and lCO did the inclusion of additional information from sibs make the posterior means of π from different chains more similar to each other.

### Information from parental average EBV

In dairy cattle, genomic predictions are often combined with pedigree information [4] before obtaining final genomic EBV. In our study, lEW was the only trait for which adding pedigree-based information significantly improved predictive ability. The increase in the R-square of the regression equation to predict hatch-corrected phenotypes from generation 5 when adding PBLUP to marker-based EBV was significant (p < 0.05) only for lEW for GBLUP and Bayes-C-π, for which the R-square increased from 0.174 to 0.189 and from 0.187 to 0.203, respectively. Increases in R-square were not significant (p > 0.05) for all other traits using both methods. This suggests that in this dataset, the markers capture most of the pedigree information, likely because all the parents were genotyped.

For most traits, the predictive ability of the marker-based EBV was not substantially lower for traits measured at a late age (Figures 1 and 2), although late traits were only recorded on selected individuals and estimated heritabilities for late traits were generally lower than for the corresponding traits measured at a younger age. This indicates that having records only on selected parents did not limit the ability to estimate marker effects.

In Figures 1 and 2, the difference between the accuracy of marker- versus pedigree-based parental average EBV (e.g. GBLUP-PA vs. PBLUP) reflects the gain in information from more accurate EBV of parents when using markers, while the difference between the accuracy of marker-based parental average EBV and marker-based individual EBV (e.g. GBLUP-PA vs. GBLUP) arises from markers providing information on Mendelian sampling terms. For ePS and ePD and eSM, the increase in accuracy at an early age could be attributed mostly to better estimates of parental EBV. For all other traits, increases in accuracy were primarily based on markers providing information on Mendelian sampling terms. For EBV for selection at a later age, the improvement originated mostly from Mendelian sampling terms, probably because the pedigree parental average EBV were much more accurate than at the earlier age.

### Reduced animal model

The reduced animal model was used to incorporate genomic information into genetic evaluation using GBLUP. It was possible to use this model here because all the parents were genotyped, thus data from non-genotyped individuals could be included without loss of information. If some parents are not genotyped, the 1-step methods that combine pedigree-based and genomic relationships can be used to avoid loss of information [7,8]. An alternative to the 1-step method is the use of deregressed EBV [5,6] but this involves approximations and a potential loss of information.

In fact, the model used here represents a special case of the 1-step method of Legarra et al. [7], where all non-genotyped individuals in the data are non-parent progeny. In this case, the only pedigree-relationships that are used are those between genotyped parents and their non-genotyped progeny. Without inbreeding, the expectation of these relationships is equal to 0.5, both based on pedigree and based on genomic data, because progeny receive half of their alleles from each parent. Thus, in this special case, combining genomic and pedigree relationships does not require the rescaling that is typically required for the 1-step approach [20]. In addition to avoiding the need for rescaling, this special case allows equations for non-parents to be absorbed, as in the reduced animal model, which reduces computational demands, although the main computational task of inverting the dense genomic relationship matrix of genotyped individuals remains. By absorbing non-parents, computing time for the reduced animal model is proportional to n^3^, where n is the number of genotyped animals, while the number of animals with phenotypes has a negligible impact on computing time. Computing time for Bayes-C-π is proportional to the number of markers and to the number of records. The reduced animal model can also easily be extended to a multi-trait setting, following standard multiple-trait animal model procedures. Finally, applying a reduced animal model makes it possible to use weighted genomic relationship matrices that accommodate differential weights on SNP, depending on their effects, similar to the Bayesian model averaging methods [21]. Use of a weighted genomic relationship matrix in a multi-trait setting, however, requires further work.

### Implementation of genomic selection in layer chickens

Increases in accuracy were evaluated when selection is at a very early age, prior to phenotypes being available on selection candidates or their sibs, and at a later age. Late age selection represents a scenario in which genomic information is used to increase accuracy of selection in existing layer breeding programs, particularly in the case of males, which are primarily evaluated based on sib information in current breeding programs. Early age selection represents a scenario in which the benefits of genomic selection are capitalized on by also reducing the generation interval from the traditional one year to half a year, as proposed by Dekkers et al. [22]. Using these results, breeding programs exploiting genomic information can be optimized, including scenarios where only male candidates are genotyped and where population sizes are reduced to capitalize on the effect of GEBV on rates of inbreeding. The use of low-density SNP panels needs to be evaluated [23] to reduce costs of genotyping, but this was beyond the scope of this research. In this study, the size of the training data was limited compared to what is available in dairy cattle and increasing its size is expected to further increase the accuracy of GEBV.

## Conclusions

Reduced animal model approaches can be used to estimate breeding values from high-density SNP data when all parents have been genotyped. Marker-based methods improve the prediction of future performances compared to the classical pedigree-based approach, with most of the accuracy increase due to improved estimation of Mendelian sampling terms. The advantage of marker-based methods is greater for selection at a young age, before information on sibs of selection candidates is available. The accuracies of methods that assume equal variance for all SNP, such as GBLUP and of those that allow differential weighting and shrinkage of SNP effects are similar.

## Competing interests

The authors declare that they have no competing interests.

## Authors' contributions

All authors conceived the study, contributed to methods and to writing the paper and also read and approved the final manuscript. AW undertook the analysis and wrote the first draft. Data were prepared by JA, PS, JF and NPO. JCMD provided overall oversight of the project.
